# Functional Outcome of Uncemented Total Hip Replacement in Low Socioeconomic Group Using Modified Harris Hip Score: A Prospective Midterm Follow-Up Study

**DOI:** 10.7759/cureus.50005

**Published:** 2023-12-05

**Authors:** Devendra Lakhotia, Utkarsh Agrawal

**Affiliations:** 1 Department of Orthopedics, Jaipur National University, Institute for Medical Sciences and Research Centre, Jaipur, IND; 2 Orthopedics, Jaipur National University, Institute for Medical Sciences and Research Centre, Jaipur, IND

**Keywords:** functional outcome, modified harris hip score, low socioeconomic group, total hip replacement, uncemented

## Abstract

Background

Total hip replacement is a safe and effective surgery with excellent outcomes in most patients with hip arthritis. The aim of this study was to evaluate functional outcomes and complications of total hip replacement among patients with low socioeconomic status in India.

Methods

We assessed 50 patients whose incomes fell below the poverty line and who underwent uncemented total hip replacement. We used a modified Harris Hip Score, replacing two items (one measuring range of motion and one measuring deformity) with two new ones (one related to return to professional activity and another regarding sexual activity).

Results

At the final follow-up, patients' modified Harris Hip Score improved from a preoperative mean value of 13.28 (0-46) to a postoperative mean value of 88.52 (64-100), suggesting marked improvement in functional outcome (p<0.001). In total, 32 (64%) patients returned to their original profession, and 12 (24%) switched to alternate work with mild pain. All patients reported satisfaction with their sexual activity at the final follow-up.

Conclusions

Many patients in India whose income is below the poverty line work in manual labor professions (e.g., farmers, masons, and cobblers) that increase their risk of hip damage. Total hip replacement is beneficial for these patients, offering good personal and professional quality of life after the surgery.

## Introduction

Total hip replacement (THR) replaces damaged and worn hip joints with a smooth, artificial prosthesis. This low-risk procedure offers improved hip function and flexibility, reduced pain, and better stability in 95% of patients [1]. However, the benefits of THR among adults with low socioeconomic status remain unclear, particularly in countries where a large proportion of the population is below the poverty line. In India, for example, many individuals face social, economic, and cultural barriers to health care, such as illiteracy, unhealthy living conditions, inadequate access to postoperative care, and higher rates of comorbidities [2-4]. Yet, there is a need for THR in these populations as much as in affluent societies, as working conditions often require squatting or cross-legged posture, leading to hip pain and dysfunction [5].

The main aim of this prospective study was to evaluate the results of uncemented THR in patients with low socioeconomic status, particularly the impact of total hip arthroplasty on personal and professional quality of life. The use of uncemented THR in this adult population is of particular interest because it may offer advantages over cemented THR, such as decreased risk of loosening and improved long-term survival of the implant. Potential disadvantages include increased early femoral loosening and increased risk of femoral fractures [6]. The results can provide important insights into the effectiveness and safety of this surgical procedure in underserved populations.

## Materials and methods

This prospective study was conducted at a tertiary care center in Rajasthan, India. The study enrolled 60 consecutive patients (89 hips) from 2008 to 2011 who had below poverty line (BPL) cards. The BPL cards were issued by the Government of India to the population of low socioeconomic status. The Indian government covers the full cost of implants and surgery for these patients.

In this study, indications for uncemented THR were avascular necrosis, post-traumatic arthritis, and inflammatory arthritis. We excluded patients older than 65 years, those with osteoporosis or a history of revision surgery, and those who were candidates for cemented arthroplasty. Clinical assessment of patients was performed using a modified Harris Hip Score (HHS). Pain and functional capacity were the two most important criteria with the highest points (44 and 47 points, respectively, out of 100 total points). Two items, one measuring range of motion (five points) and one measuring deformity (four points), were removed. Two measures were added: one related to sexual activity (Table 1) and one related to socioeconomic (professional) quality of life (Table 2).

**Table 1:** Grading sexual satisfaction due to hip pathology

Description	Grade	Points
Able to perform with no pain, difficulty and fear, fully satisfied	A	5
Able to perform with no pain, difficulty and fear, satisfied	B	4
Able to perform with mild pain, difficulty and fear, satisfied	C	3
Able to perform with moderate pain, difficulty and fear, partially satisfied	D	2
Able to perform with pain, difficulty and fear, not satisfied	E	1
Not able to perform	F	0

**Table 2:** Grading professional performance after hip replacement surgery

Description	Class	Points
Return to original profession with satisfaction and no pain	I	4
Return to alternate profession with satisfaction and no pain	II	3
Return to alternate profession with mild pain and difficulty, partially satisfied	III	2
Return back to profession with moderate to severe pain, not satisfied	IV	1
Not working at all	V	0

All surgeries were performed by a senior joint replacement surgeon using a posterior Moore approach [7], with the patient in a lateral position. The prosthesis used was an uncemented metal-on-poly implant with a proximally porous-coated femoral stem and 32-36 mm head, depending on the size of the acetabulum liner. All participants were assigned a modified HHS at three months, six months, 12 months, and then yearly postoperatively for five years. All patients were allowed full weight bearing with support on the first postoperative day, and all patients were walking full weight bearing without support at three months follow-up.

All data were recorded and analyzed using SPSS v. 21 (IBM Inc., Armonk, US). Student's t-test and Fisher's exact test were used to determine significance.

## Results

Out of 60 patients, 10 patients were lost to follow-up. So, 50 patients (76 hips; 24 unilateral and 26 bilateral) were registered at the final follow-up. All 50 patients were followed up for at least five years. The average follow-up period was 74.2 months, with a minimum of 60 months and a maximum of 96 months. Out of 50 patients, 34 (68%) were male and 16 (32%) were female. The mean age of the cohort was 43.24 years (range 20 to 65 years). The most common etiology was avascular necrosis (80%), followed by post-traumatic secondary arthritis (12%), rheumatoid arthritis (4%), and ankylosing spondylitis (4%). Among the 50 patients, eight patients worked as laborers (16%), 14 farmers (28%), two cobblers (4%), two painters (4%), and two heavy motor vehicle drivers (4%). All 16 (32%) female participants performed household and farm chores. The remaining six patients (12%) were in sedentary jobs with light work.

Four patients had superficial wound complications at the surgical site managed with debridement and secondary suturing. No patient developed a serious infection. The average limb length discrepancy was <0.5 cm, with two cases exhibiting a limb length discrepancy exceeding 2 cm. Sciatic nerve palsy was present in one case and only partially recovered at the five-year follow-up.

The mean modified HHS improved from 13.28 (0-46) preoperative to 88.52 (64-100) at the final follow-up (minimum five years of follow-up), which suggests marked improvement (p<0.001) in function (Table 3). Excellent to good results were found in 80% of patients, with only four percent (two cases) graded as poor and the rest graded as fair.

**Table 3:** Modified Harris Hip score for pain and function

Elements	Mean preoperative value (range)	Mean postoperative value at final follow-up (range)	p-value
Pain	4.40 (0-20)	40.264 (30-44)	<0.001
Limp	0.20 (0-5)	9.20 (8-11)	<0.001
Support	3.44 (0-11)	10.36 (5-11)	0.004
Walking distance	1.12 (0-5)	9.6 (2-11)	0.001
Sitting	0.84 (0-3)	4.28 (3-5)	0.003
Stairs	0.44 (0-1)	3.48 (1-4)	0.002
Public transportation	0.40 (0-1)	1 (0-1)	0.01
Shoes and socks	0.88 (0-2)	3.68 (2-4)	0.004
Sexual satisfaction	1.1 (0-2)	3.28 (3-5)	<0.001
Housework	0.40(0-1)	3.44 (3-4)	0.002
Professional activities	0.7 (0-1)	3.04 (2-4)	<0.001
Harris Hip Score	13.28 (0-46)	88.52 (64-100)	<0.001

At the final follow-up, 32 (64%) patients had returned to their original professions, 12 (24%) had switched to alternate work with mild pain and difficulty, and six (12%) had not returned to their previous or any profession. All female patients returned to their original housework with some modification in sitting: 44% within four months postoperatively, 31% within six months, and the rest within one year. Prior to THR, 60% of patients were disabled, 36% had marked pain, and four percent had moderate pain. At the final follow-up, 92% of patients had either no pain or slight pain, and the remaining eight percent had mild pain.

## Discussion

We assessed 50 low-income patients who underwent THR at a hospital in India for their ability to resume their routine and professional activities. We used a modified HHS to evaluate various aspects of their professional and personal quality of life. The original HHS was developed by WH Harris in 1963 to measure hip pain and function on a 100-point scale [8]. In our modified version, we retained the original measures for pain (44 points) and function (47 points), but we dropped the measures for deformity and range of motion. These measures were less relevant for our patient population, and research has shown that surgeon-based outcome assessments are prone to bias [9]. Increasing evidence shows that patient-based outcome measures are more reliable than those based on clinical scores [10]. Thus, we included self-reported sexual activity (up to five points) and returning to one's original profession or duties (up to four points).

Other studies have similarly modified the HHS to make it more relevant for the studied population [10,11]. For example, Kocic et al. [12] added measures related to rising from a chair and bathing. We evaluated sexual satisfaction as our patient population skewed relatively younger, with a mean age of about 43 years. To our knowledge, no other scoring system has included this criterion, and we felt it was important in our setting, where discussion of sexual activity is often considered taboo. We found patients were responsive to this line of questioning.

Hip pathology in 80% of our patients was avascular necrosis of the femoral head, also likely due to the relatively younger age group (mean age 43.24 years). Primary osteoarthritis of the hip joint is mainly due to age-related wear and tear and is rare in India. Due to gaps in access and affordability of health care in India, low-income individuals often seek advice from unqualified medical practitioners who prescribe drugs like steroids and other treatments with adverse long-term effects [13]. These populations also are vulnerable to alcohol and tobacco use, which can exacerbate symptoms.

The mean modified HHS increased from 13.28 (range 0-46) preoperatively to 88.52 (range 64-100) postoperatively at the final follow-up, an improvement equivalent to results observed in other studies [12,14], despite the lower socioeconomic status and education levels of patients in our study. Nearly all (96%) patients were satisfied with the surgery, and scores ranged from excellent to good for 80% of them. Specifically, for those with a preoperative modified HHS <20, 57% attained excellent results, and for those with a preoperative HHS of 21-40, 60% attained excellent results, indicating no correlation between the pre- and postoperative modified HHS (p=0.687). Preoperative function is strongly associated with both long- and short-term functional outcomes [15]. This finding may explain why patients in our study, who were younger with good muscle mass and fewer comorbidities, fared better even if their preoperative modified HHP was lower.

The average score among those whose preoperative symptoms lasted fewer than three years was 93.5, compared to 79.67 for patients whose symptom history exceeded three years (p=0.0025). Prolonged osteoarthritic symptoms may result in muscle atrophy, tissue contracture, and unrecoverable deterioration of the patient's general condition [16]. Therefore, the timing of THR has clinical significance for patients in low socioeconomic groups, with earlier treatment associated with better outcomes.

Compared to results from previous studies [17,18], our results revealed improved sexual function after THR. Our study population's younger age again may be a factor. Compared to older patients, younger patients may have lower psychological inhibition and engage more in sexual activity. Counseling for normal sexual activities after the surgery helped alleviate patients' fear about damage to the artificial hip joint. Also, our participants had no etiology of rheumatoid arthritis or co-morbid musculoskeletal problems, which can adversely affect sexual function. Our results further indicated that the level of education did not affect HHS outcomes. This finding refutes previous findings, such as those of Young et al. [19], who found that patients with at least a high school education scored higher postoperatively than those with less than a high school education.

No patients in our study developed deep infections, wound gaps, bedsores, or dislocation. Four developed a superficial infection within six weeks of surgery that healed with debridement and secondary suturing. One patient with sciatic nerve palsy only partially recovered at the final follow-up. This patient had a poor functional outcome and dissatisfaction with the surgery due to complete motor and sensory deficit. The posterior approach and use of an uncemented femoral stem are risk factors for sciatic nerve palsy [20].

In our study, we found no evidence that social, educational, and occupational factors serve as predictors of short and midterm outcomes of THR. In contrast, Schäfer et al. [21] evaluated social, educational, and occupational predictors of THR outcomes and found that manual laborers had lower postoperative scores in comparison to employed and self-employed patients. Their results indicate that health services may want to identify patients in need of special attention to increase their respective postoperative scores.

Overall, 96% of patients in our study were satisfied with their THR results at the five-year follow-up. Mancuso et al. [22] reviewed patient expectations and satisfaction with total hip arthroplasty and concluded that approximately 90% were satisfied, and patient satisfaction was mainly influenced by patients' preoperative expectations, objective postoperative outcomes, and preparation before surgery. Among those dissatisfied, pain is the chief complaint [23]. In our study, most of the patients (92%) had either no or slight pain. This superior outcome may reflect realistic expectations among patients in this socioeconomic group.

We acknowledge some limitations of our study. The small sample size, though representative of THR incidence in the studied population, may hinder the generalizability of our results. Although this study evaluated midterm outcomes (minimum five years of follow-up), longer-term evaluation in a larger sample size may provide more meaningful conclusions in this group of patients. In addition, radiographic analysis was not conducted to correlate the clinical score. However, the main objective to evaluate the functional outcomes of THR in patients with low socioeconomic status, as well as the benefits and risks of the procedure, was fulfilled.

## Conclusions

In our study, we found good to excellent functional outcomes, with minimal risk and complications in most cases. Most patients returned to their same profession; only a few had to switch professions due to post-surgical behavior modifications. Most patients also were satisfied with their sexual activity after surgery. To conclude, total hip replacement is beneficial in low socioeconomic group patients, offering good personal and professional quality of life after the surgery.
